# Postbreeding elevational movements of western songbirds in Northern California and Southern Oregon

**DOI:** 10.1002/ece3.3326

**Published:** 2017-08-23

**Authors:** Andrew Wiegardt, Jared Wolfe, C. John Ralph, Jaime L. Stephens, John Alexander

**Affiliations:** ^1^ Klamath Bird Observatory Ashland OR USA; ^2^ Redwood Sciences Laboratory USDA Forest Service, Pacific Southwest Research Station Arcata CA USA

**Keywords:** altitudinal movements, birds, breeding, mist net, molt migration, Pacific Northwest, passerines

## Abstract

Migratory species employ a variety of strategies to meet energetic demands of postbreeding molt. As such, at least a few species of western Neotropical migrants are known to undergo short‐distance upslope movements to locations where adults molt body and flight feathers (altitudinal molt migration). Given inherent difficulties in measuring subtle movements of birds occurring in western mountains, we believe that altitudinal molt migration may be a common yet poorly documented phenomenon. To examine prevalence of altitudinal molt migration, we used 29 years of bird capture data in a series of linear mixed‐effect models for nine commonly captured species that breed in northern California and southern Oregon. Candidate models were formulated a priori to examine whether elevation and distance from the coast can be used to predict abundance of breeding and molting birds. Our results suggest that long‐distance migrants such as Orange‐crowned Warbler (*Oreothlypis celata*) moved higher in elevation and Audubon's Warbler (*Setophaga coronata*) moved farther inland to molt after breeding. Conversely, for resident and short‐distance migrants, we found evidence that birds either remained on the breeding grounds until they finished molting, such as Song Sparrow (*Melospiza melodia*) or made small downslope movements, such as American Robin (*Turdus migratorius*). We conclude that altitudinal molt migration may be a common, variable, and complex behavior among western songbird communities and is related to other aspects of a species’ natural history, such as migratory strategy.

## 1 INTRODUCTION

Long‐distance molt migration is a mechanism by which migratory species deal with the energetic demands of the postbreeding molt (definitive prebasic molt sensu Wolfe et al., 2014) by moving to seasonally food‐rich environments to replace their body and flight feathers (Pyle et al., 2009). Even at smaller spatial scales, resident and facultative migratory birds must acquire the dispersed and seasonal food resources necessary for successful completion of postbreeding molt (Daan et al., 1988; Murphy & King, 1992). Seasonal food resources are particularly patchy in mountainous areas where insect and fruit abundance can vary dramatically across relatively short distances (Thomas, 2005). As such, altitudinal molt migration should be expected in many species that breed and molt in montane areas.

Molt is an energetically costly process necessary for the maintenance of feathers and plumage. As such, birds may suffer from a limited capacity to thermoregulate (Schieltz & Murphy, 1997) or sustain flight (Hedenström & Sunada, 1999) during molt. These limitations make birds more susceptible to the deleterious effects of inclement weather, or the inability to escape predators. The relatively early timing of Western Sandpiper (*Calidris mauri*) molt migration has been suggested as an adaptation to avoid a common migratory predator, the Peregrine Falcon (*Falco peregrinus*, Lank et al. 2003). The vulnerability of molting birds to predation and inclement weather may result in increased mortality, which in‐turn can affect population viability (Swaddle & Witter, 1997). In addition to potential direct demographic effects, nonlethal events during the molting season may have indirect carry‐over effects on other phases of the avian life cycle, such as breeding (Slagsvold & Dale, 1996). As such, the timing of molt is most likely highly adaptive and particularly susceptible to changes driven by natural selection. For example, western and eastern populations of Warbling Vireos (*Vireo gilvus*) molt on the winter and summer grounds, respectively (Pyle, 1997); such differences presumably reflect local adaptation and aid in the successful completion of molt across longitudes. To better understand selective pressures responsible for differences in molt strategies and the influence of lethal and nonlethal effects experienced during molt on population viability, we first need to determine when and where birds molt, and identify those landscape features associated with molt.

To date, few studies have endeavored to associate landscape features with altitudinal molt migration in the western United States. However, the limited number of studies that examined altitudinal molt migration in the western United States suggests a general pattern of upslope movements after breeding to undergo molt. For example, Rowher, Rowher, and Barry (2008) used point counts conducted in the spring and fall and found that Cassin's Vireo (*Vireo cassinii*) were more abundant at higher elevations during the fall molting season than in the spring. Steele and McCormick (1995) captured birds at several different elevations in the Sierra Nevada Mountains of California and observed adults leaving the breeding grounds at lower elevations and moving to higher elevation sites, where they had not been captured during the breeding season, to undergo molt. Presumably, many birds move upslope to wet montane meadows during the molting season to take advantage of insect food resources (Van Dyke, 1919).

To examine relationships between landscape features and postbreeding movements prior to molt, we used data from a network of banding stations for species known to molt on their summer grounds. Specifically, we examined relationships between abundances of breeding and molting birds and landscape features such as elevation and distance from coast. In total, we examined nine of the most commonly captured species in northern California and southern Oregon.

Studies suggest that higher elevation habitats retain more moisture, relative to lower elevations, during hot and dry late‐summer and early fall periods throughout our study area (Patton & Judd, 1970; Robinson et al., 2013). We suspect that insect abundance, an important food resource for molting birds, is strongly correlated with moisture during these hot and dry periods (sensu Van Dyke, 1919). We formulated three a priori hypotheses regarding movements of western passerines between periods of breeding and molting. (i) We hypothesize that some species of western passerines move across elevations after the breeding season to complete molt. If our first hypothesis is correct, we expect that there will be higher abundances of molting birds at higher elevations and further from the coast when compared to abundances of breeding birds. (ii) Long‐distance migrants are physiologically adapted to move great distances; therefore, we hypothesize that many long‐distant migrant species have evolved to seek far‐off habitats with greater resources during postbreeding molt. If this is true, we expect to find spatially disparate populations of breeding and molting long‐distant migrant birds throughout our study area. (iii) Conversely, we hypothesize that resident birds are less equipped to make postbreeding movements, which would result in less‐distant and adjacent populations of breeding and molting resident species throughout our study area.

## 2 METHODS

**Figure 1:**
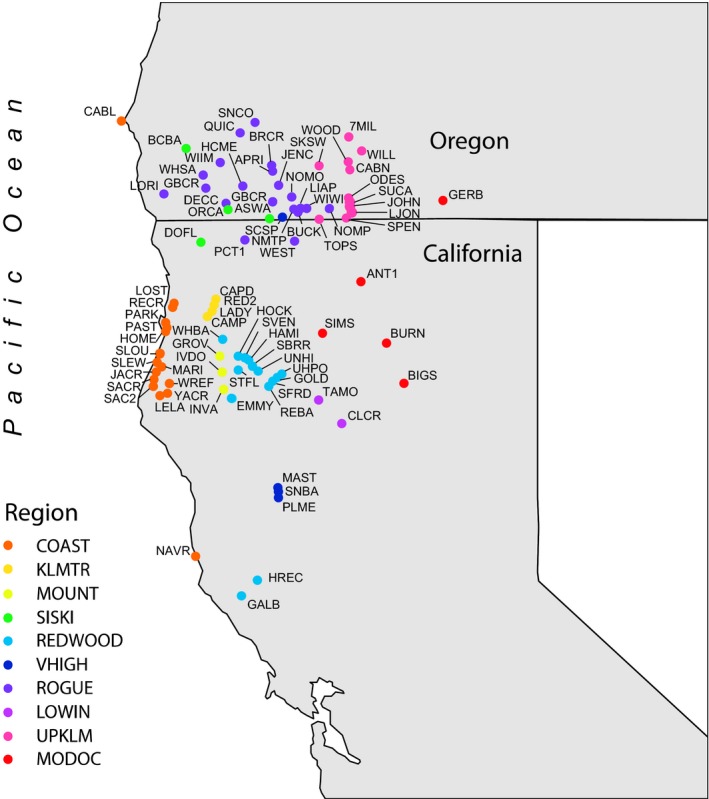
Map of southern Oregon and northern California showing the locations of 82 banding stations where adult birds were observed breeding or molting, color coded by region. Information on the elevation, latitude, and longitude of each station can be found in AppendixA

To test the aforementioned hypotheses, we measured differences in the abundance of breeding and molting birds relative to migratory guild, elevation, and distance from coast using mist‐netting data from 82 stations in the Klamath‐Siskiyou Bioregion of northern California and southern Oregon from 1982 to 2011 (Figure 1, Appendix A). Each station was operated from 2 to 27 years, and the average length each station was operated is 9.2 years. We operated two banding stations year‐round; all others were operated from April through October. Each banding station was scheduled at least once every 10 days during months of operation. Each station had eight to 15 net sites that were opened 15 min prior to sunrise and operated for 5–6 hr during each sampling day. For complete information on the banding methodology, see Alexander, Ralph, Hollinger, and Hogoboom (2004). Birds were aged and sexed following Pyle (1997), and other morphometrics were obtained following Ralph, Geupel, Pyle, Martin, and Desante (1993). Study species were chosen based on their abundance and diversity of migratory behaviors. For some species, (e.g., Song Sparrow) it was difficult to determine whether populations in our study area are resident, short‐distance, or altitudinal migrants. Therefore, we lumped study species into two distinct migratory groups: resident/short‐distance migrants and long‐distance Neotropical migrants. Study species included five resident/short‐distance migrants: Spotted Towhee (*Pipilo maculatus*), Wrentit (*Chamaea fasciata*), American Robin (*Turdus migratorius*), Oregon Dark‐eyed Junco (*Junco hyemalis oreganus*), and Song Sparrow (*Melospiza melodia*); and four long‐distance migrants: MacGillivray's Warbler (*Geothlypis tolmiei*), Orange‐crowned Warbler (*Oreothlypis celata*), Audubon's Yellow‐rumped Warbler (*Setophaga coronata auduboni*), and Swainson's Thrush (*Catharus ustulatus*).

To measure changes in the abundance of breeding birds and those in postbreeding molt, we included only adult birds aged as after hatching year according to Pyle (1997a). We classified individuals as breeding if they had vascularized or wrinkled brood patches or cloacal protuberances that were medium or large (following Ralph et al., 1993). We classified individuals as molting if they were captured growing flight feathers symmetrically. We removed recaptures of the same individual within a season to avoid pseudoreplication. We standardized abundance of breeding and molting birds by individual captures per 100 net hours per station.

For each species, we created two candidate model sets of 16 zero‐inflated linear regression models with a Poisson distribution using R (R Core Team 2015) and the glmmADMB package (Fournier et al., 2012). The response variables for the two candidate model sets were either the standardized abundance of breeding birds of a particular species or the standardized abundance of molting birds of a species. Due to the nature of the banding stations being operated some years and not others and the various durations each station was run, we felt that modeling species abundance by each sampling day coupled with a zero‐inflated Poisson distribution was the best way to overcome unequal sampling effort while still maintaining our large sample size. Explanatory variables were elevation (meters), quadratic elevation (meters), distance from coast (kilometers), and quadratic distance from coast (kilometers). Julian date (day of the year) was included in every model as a nuisance parameter. Furthermore, we delineated the study area and aggregated banding stations into 10 regions based on similarities in geography (Table 1). We compared the inclusion of a random intercept effect associated with region to see whether it improved model fit. For each model, we examined residual plots for heteroscedasticity following Zuur, Ieno, Walker, Saveliev, and Smith (2009).

**Table 1:** Eighty‐two banding stations were grouped into ten regions defined by distance from the coast, elevation, and latitude

Region	Distance Inland (km)	Elevation (m)	Latitude
Coast	<23	>45	39.1973–42.8329
Klamath‐Trinity Rivers	40–45	110–120	41.2604–41.2957
Inland Valley	140–155	90–145	40.3069–40.5039
High‐Elevation Mountains	80–115	<1,840	39.7277–42.0331
Modoc County	145–265	930–1,660	40.6431–42.1733
Trinity Mountains	50–90	1,175–1,495	40.5161–40.9562
Redwood Forest	25–115	290–550	38.8661–40.7457
Rogue Basin	20–140	245–865	41.8325–42.8327
Siskiyou Mountains	40–127	1,205–1,635	41.8236–42.6082
Upper Klamath Lake	155–210	960–1,575	42.0262–42.7050

These regions were used for the random intercept effects in candidate models. A complete list of the stations and associated region can be found in Appendix A.

We ranked all candidate models using Akaike Information Criterion (AIC) and interpreted results based on the inclusion of explanatory variables. We also examined explanatory variable beta estimates and associated 95% confidence intervals to assess effect sizes of covariates on abundance of breeding and molting study species. Ranking models based on their AIC scores allowed us to estimate a type I error (false positive) rate for each candidate model where the highest false positive rate among top models for the 18 candidate model sets was 0.035; the overall mean type I error rate for top models was 0.006. We found colinearity between elevation and distance from coast in our dataset; therefore we did not include both covariates in any candidate single model. For top models that included a quadratic covariate as an explanatory variable, we maximized the abundance predicted by the quadratic model to determine at what elevation or distance from coast breeding and molting birds were at their highest abundance.

Post hoc, we examined each study species’ timing of the definitive prebasic molt to be certain that months of station operation (May to October) captured the breadth of breeding and molting activity. We have two banding stations (HOME and WIWI) that were run year around and would bias our results if study species were found breeding or molting before May or after October at these stations.

## 3 RESULTS

During the course of the study, we captured 37,886 breeding and postbreeding molting adult birds of the nine study species (Table 2). Of these, Song Sparrow (*n* = 12,877) and Orange‐crowned Warbler (*n* = 1,552) were our most common and least common study species, respectively.

**Table 2:** Total number of individuals for each of our nine study species captured in breeding or molting condition and their migratory guild, in the Klamath‐Siskiyou Bioregion in northern California and southern Oregon

Common name	Species code	Total breeding	Total molting	Migratory guild
MacGillivray's Warbler	MGWA	3,541	655	Long‐distance
Swainson's Thrush	SWTH	4,068	541	Long‐distance
Wrentit	WREN	1,919	441	Resident/short‐distance
Oregon Junco	ORJU	3,072	2,160	Resident/short‐distance
American Robin	AMRO	2,401	452	Resident/short‐distance
Audubon's Warbler	AUWA	1,340	493	Long‐distance
Spotted Towhee	SPTO	1,780	594	Resident/short‐distance
Song Sparrow	SOSP	9,088	3,789	Resident/short‐distance
Orange‐crowned Warbler	OCWA	862	690	Long‐distance

**Figure 2:**
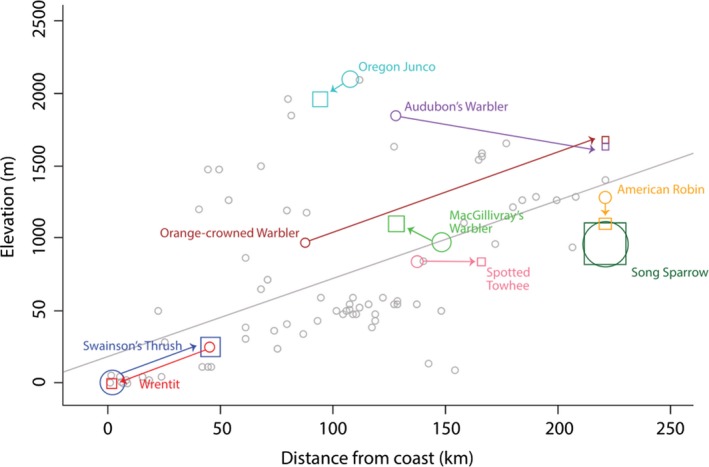
Scatterplot showing elevation (m) and distance from coast (km) of each banding station (gray circles) and regression line (gray) of station‐elevation predicted by distance from the coast. Colored arrows represent shifts in peak abundance of breeding (colored circles) and molting species (colored squares) as determined by maximizing a quadratic linear model with either elevation or distance from the coast. Sample sizes (individuals per 100 net hours) are indicated by the relative sizes of colored squares and circles

All long‐distance migrant species showed a significant molt migration movement upslope or further inland after the breeding season (Figure 2). For example, MacGillivray's Warblers exhibited a quadratic elevation covariate in the top model for both breeding (Table 3) and molting abundances (Table 4). Based on the top models from the two candidate model sets (breeding and molting; Appendix B), we found that estimated peak abundance of breeding MacGillivray's Warblers was at 964 m elevation (β_1_ = −0.997, β_2_ = 3.199, *z*_1_ = −6.35, *z*_2_ = 10.97, 95% CI of β_1_ (−0.689, −1.305), 95% CI of β_2_ (2.626, 3.769); Figure 2), while the estimated peak abundance of molting MacGillivray's Warblers was slightly, but significantly, higher at 1,096 m (β_1_ = −1.026, β_2_ = 2.562, *z*_1_ = −6.35, *z*_2_ = 10.97, 95% CI of β_1_ (−1.683, −0.370), 95% CI of β_2_ (1.415, 3.708); Figure 2).

**Table 3:** A table summarizing the top model and null model for each species during breeding with associated delta AIC, number of parameters (*K*), *p*‐value of the beta estimate of the elevation or distance from the coast parameter, and AIC Weights for each model

Species	Model	∆AIC	*K*	*p*‐value	AIC weights
Audubon's Warbler	Elevation2	n/a	13	<.001	1.00
	Null	603	1	n/a	0
MacGillivray's Warbler	Elevation2	n/a	13	<.001	1.00
	Null	620	1	n/a	0
Orange‐crowned Warbler	Elevation	n/a	12	<.001	0.68
	Null	140	1	n/a	0
Swainson's Thrush	Distance from coast2	n/a	13	<.001	1.00
	Null	1,211	1	n/a	0
Oregon Junco	Elevation2	n/a	13	<.001	1.00
	Null	1,053	1	n/a	0
Spotted Towhee	Elevation2	n/a	13	<.001	1.00
	Null	532	1	n/a	0
American Robin	Elevation2	n/a	13	<.001	0.99
	Null	541	1	n/a	0
Song Sparrow	Distance from coast2	n/a	13	<.001	1.00
	Null	1,117	1	n/a	0
Wrentit	Distance from coast2	n/a	13	<.001	1.00
	Null	174	1	n/a	0

A “2” at the end of the model name denotes a quadratic model. All models included Julian date as a nuisance parameter. All of the top models included a random intercept effect of region. The complete AIC tables with all 16 candidate models for each candidate model set can be found in Appendix B.

**Table 4:** A table summarizing the top model and null model for each species during molt with associated delta AIC, number of parameters (*K*), *p*‐value of the beta estimate of the elevation or distance from the coast parameter, and AIC weights

Species	Model	∆AIC	*K*	*p*‐value	AIC weights
Audubon's Warbler	Elevation2	n/a	13	.035	1.00
	Null	989	1	n/a	
MacGillivray's Warbler	Elevation2	n/a	13	<.001	0.99
	Null	113	1	n/a	
Orange‐crowned Warbler	Elevation	n/a	12	.035	0.99
	Null	203	1	n/a	
Swainson's Thrush	Distance from coast2	n/a	13	<.001	0.99
	Null	127	1	n/a	
Oregon Junco	Elevation2	n/a	13	<.001	1.00
	Null	960	1	n/a	
Spotted Towhee	Elevation2	n/a	13	<.001	0.99
	Null	413	1	n/a	
American Robin	Elevation2	n/a	13	.001	0.87
	Null	158	1	n/a	
Song Sparrow	Distance from coast2	n/a	13	.018	0.99
	Null	461	1	n/a	
Wrentit	Distance from coast2	n/a	13	<.001	0.85
	Null	253	1	n/a	

A “2” at the end of the model name denotes a quadratic model. All models included Julian date as a nuisance parameter. All of the top models included a random intercept effect of region. The complete AIC tables with all 16 candidate models for each candidate model set can be found in Appendix B.

In contrast, Swainson's Thrushes exhibited a quadratic distance from the coast covariate in the top model for both breeding (Table 3) and molting (Table 4). We found that breeding Swainson's Thrushes were closer to the coast (β_1_ = −2.23 × 10^−4^, β_2_ = 0.026, *z*_1_ = −13.39, *z*_2_ = 10.50, 95% CI of β_1_ (−1.91 × 10^−4^, −2.55 × 10^−4^), 95% CI of β_2_ (0.021 0.031)), while molting individuals were further inland (β_1_ = −2.68 × 10^−4^, β_2_ = 0.039, *z*_1_ = −5.81, *z*_2_ = 4.81, 95% CI of β_1_ (−1.78 × 10^−4^, −3.59 × 10^−4^, 95% CI of β_2_ (0.023, 0.055)). Specifically, the breeding peak abundance of Swainson's Thrush was found 1 km inland from the coast but 45 km inland from the coast during molt (Figure 2).

For other long‐distance migrants, Audubon's Warblers and Orange‐crowned Warblers, covariates in the top model differed between breeding and molting birds. That is, for both Audubon's Warbler and Orange‐crowned Warbler, the top candidate model included elevation when individuals are breeding, and distance inland when they are molting (Tables 3 and 4). We estimated a peak of breeding Audubon's Warblers at 1,846 m elevation (β_1_ = −3.783, β_2_ = 9.524, *z*_1_ = −7.21, *z*_2_ = 7.19, 95% CI of β_1_ (−4.811, −2.754), 95% CI of β_2_ (6.942 12.143)) with higher abundances of molting birds further inland (β_1_ = −1.60 × 10^−5^, β_2_ = 0.016, *z*_1_ = −0.64, *z*_2_ = 2.11, 95% CI of β_1_ (−6.44 × 10^−5^, 3.28 × 10^−5^), 95% CI of β_2_ (−1.09 × 10^−3^, 0.030)). The peak abundance of breeding warblers was at 128 km from the coast, while molting birds were 221 km inland (Figure 2). Orange‐crowned Warblers were found to molt at higher elevations (β* * = 0.931, *z* = 5.55, 95% CI of β (0.455 1.408)) averaging 900 m while breeding and 1,500 m while molting. They also were found to molt further from the coast (β_1_ = 5.50 × 10^−5^, β_2_ = 0.010, *z*_1_ = −3.11, *z*_2_ = 2.11, 95% CI of β_1_ (−2.02 × 10^−5^, −8.95 × 10^−5^), 95% CI of β_2_ (7.2 × 10^−4^, 0.020)), as breeding Orange‐crowned averaged at 87 km inland (Figure 2) while molting birds were at 221 km.

Resident/short‐distance migrant species generally did not show notable molt migration movements, either upslope or further inland, between breeding and molting (Figure 2). Three resident/short‐distance migrant species had elevation in the top model for both breeding (Table 3) and molting abundances (Table 4). American Robin showed a difference in the elevation of breeding birds at 1,296 m (β_1_ = −0.768, β_2_ = 2.221, *z*_1_ = −2.76, *z*_2_ = 3.38, 95% CI of β_1_ (−0.222, −1.313), 95% CI of β_2_ (0.934, 3.507); Figure 2) and molting birds slightly lower at 1,096 m elevation (β_1_ = −0.810, β_2_ = 1.684, *z*_1_ = −2.68, *z*_2_ = 3.24, 95% CI of β_1_ (−1.402, −0.218), 95% CI of β_2_ (0.665, 2.702); Figure 2). Similarly, breeding Oregon Juncos’ peak abundance was at 2,087 m (β_1_ = −2.604, β_2_ = 8.260, *z*_1_ = −8.35, *z*_2_ = 9.99, 95% CI of β_1_ (−1.993, −3.214), 95% CI of β_2_ (6.639, 9.881); Figure 2), while the peak abundance of molting birds was lower at 1,954 m (β_1_ = −5.049, β_2_ =14.363, *z*_1_ = −15.1, *z*_2_ = 14.1, 95% CI of β_1_ (−4.346, −5.751), 95% CI of β_2_ (12.503, 16.224); Figure 2). For Spotted Towhees, our models suggested elevation was useful for predicting abundance of breeding and molting, with the maximum predicted abundances at 837 m during breeding (β_1_ = −2.085, β_2_ = 3.495, *z*_1_ = −8.41, *z*_2_ = 8.45, 95% CI of β_1_ (−1.599, −2.572), 95% CI of β_2_ (2.684, 4.307) and 837 m while molting (β_1_ = −1.479, β_2_ = 2.061, *z*_1_ = −4.41, *z*_2_ = 3.52, 95% CI of β_1_ (−0.822, −2.136), 95% CI of β_2_ (0.913, 3.209). For Song Sparrows, while distance to coast was in the top model for predicting both breeding (Table 3) and molting abundances (Table 4), there was no detected difference in distance to coast for peak molting and breeding bird abundance. Specifically, peak abundance of breeding (β_1_ = (3.92 × 10^−5^, β_2_ = −5.70 × 10^−3^, *z*_1_ = −8.35, *z*_2_ = 9.99, 95% CI of β_1_ (−2.69 × 10^−5^, 5.14 × 10^−5^), 95% CI of β_2_ (−2.72 × 10^−3^, −8.68 × 10^−3^)) and molting (β_1_ = 3.95 × 10^−5^, β_2_ = −5.73 × 10^−3^, *z*_1_ = 4.16, *z*_2_ = −2.37, 95% CI of β_1_ (2.09 × 10^−5^ 5.81 × 10^−5^), 95% CI of β_2_ (−9.92 × 10^−4^, −0.010)) birds were both 221 km from the coast (Figure 2). Wrentits were the only short‐distance migrant for which the top model differed between breeding and molting abundances, as they included elevation during breeding and distance inland during molt (Tables 3 and 4). The highest predicted abundance of breeding Wrentits was 45 km from the coast and at 246 m elevation (β_1_ = 1.53 × 10^−4^, β_2_ = 0.012, *z*_1_ = −5.23, *z*_2_ = 2.67, 95% CI of β_1_ (−9.56 × 10^−5^, −2.10 × 10^−4^), 95% CI of β_2_ (3.28 × 10^−3^, 0.021); Figure 2), while peak predicted abundance of molting Wrentits was very close to the coast at 1 km inland and at 3 m elevation (β* *= −4.488, *z* = −6.17, 95% CI of β (−3.062, −5.914); Figure 2). Post hoc, we found that the molting period for Wrentits extended well into November, thus biasing results of the molt models toward lower elevations and closer to the coast.

## 4 DISCUSSION

Four of the nine study species exhibited greater abundances of molting birds at higher elevations and further from the coast when compared to breeding season abundances. These results support our first hypothesis that birds commonly move to higher elevations to molt following breeding. We also found support for our second and third hypotheses that long‐distant migrants move greater distances to molt following breeding when compared to resident/short‐distant migrant birds.

Candidate models that included quadratic terms ranked better than candidate models without quadratic terms for nearly all species, suggesting that intermediate elevations and distances from the coast had higher abundances than elevations or distances at low and high extremes for most species. The exceptions were breeding Orange‐crowned Warblers and molting Wrentits. Orange‐crowned Warbler appears to have the greatest breeding abundance at higher elevations. In contrast, Wrentits appear to have the greatest molting abundances at lower elevations. The random effect of region improved model fit for all species suggesting that abundances are clustered, and geographically similar stations tend to have similar abundances of passerines. Given collinearity between elevation and distance to coast, we suspect that resident birds make postbreeding altitudinal movements over shorter distances while long‐distance migrants may also move altitudinally, but do so over longer distances, leading to elevation and distance to coast being routinely included in the top model for molting short‐ and long‐distance migrants, respectively.

Our results further support the premise that long‐distance migrants move upslope or inland to molt after breeding. Such movements may be driven by patchily distributed food resources, which require birds to follow food resources to successfully complete molt (Borgmann, Peterson, Levey, & Greenberg, 2004). Because long‐distance migrants make annual journeys across large landscapes, it follows that they are better equipped to make postbreeding movements when compared to resident birds. For example, Audubon's and Orange‐crowned warblers are long‐distant migrants that showed similar patterns with regard to movements after breeding. Although these species bred at different elevations—Audubon's Warblers at mid elevations (900–1900 m) and Orange‐crowned Warblers across a wider range of elevations (sea level to 1900 m)—both species show a shift in peak predicted abundances of over 100 km (Figure 2). Our results are concordant with Steele and McCormick (1995) where Orange‐crowned Warblers were documented leaving breeding sites at lower elevations to molt at higher ones in the Sierra Nevada Mountains.

In general, notable movements away from the breeding grounds to undergo molt were not apparent for resident/short‐distance migrant species. Our results suggest slight downslope movements for American Robins and Oregon Juncos, contrasting with the upslope and inland movements of long‐distance migrants. Populations of American Robins in California and Oregon are thought to move short distances to nearby areas after the breeding season (Vanderhoff, Sallabanks, & James, 2014). Wheelwright (1986) found that American Robins consume an approximately even mix of invertebrates and fruits during the summer, but during the fall and winter their diets become more frugivorous. Blackberries and other fruit‐bearing plants are thought to flower and fruit sooner at higher elevations and further from the coast (Sallabanks, 1993). Thus, our results tentatively support the idea that American Robins track fruit resources at lower elevations and nearer the coast in the fall. Oregon Juncos are thought to be an altitudinal migrant, with high‐elevation breeding populations moving downslope to winter. Indeed Nolan et al. (2002) have documented Dark‐eyed Juncos molting while they undergo their altitudinal migration. The timing of molt has direct and indirect effects on survival and subsequent reproduction, thereby making this trait very evolutionarily liable. It follows that there are many intermediate strategies that different species of western forest birds have developed in order to maximize survival during the molting period.

Our results suggest that Wrentits moved slightly downslope after breeding to molt. However, post hoc analysis suggested that many individuals did not finish their prebasic molt until late November at low elevations, later than nearly all inland stations. Because most banding stations were run from May through October, it is likely that the apparent downslope movement was driven by one low elevation site (HOME) that was operated year around.

We found evidence that Song Sparrows and Spotted Towhees remain on the breeding grounds during their postbreeding molt. Song Sparrows exhibit complex migratory behaviors where some individuals are thought to remain on territories year‐round while others undertake altitudinal migration (Davis & Arcese, 1999). Differences in these behaviors may reflect a gradient of adaptations to inclement weather during the fall and winter, where coastal breeders are more prone to remain on territories, as compared to high‐elevation breeders who are more likely to move downslope in response to weather or available food resources. Resident and short‐distance migrant species breeding at lower elevations, such as Song Sparrow and Spotted Towhee are more likely to stay at low elevations, where conditions during the fall and winter are more favorable than higher elevations. This strategy contrasts with that of other resident and short‐distance species, such as American Robin and Oregon Junco, which breed at higher elevations then move downslope to lower elevations where the climate is more agreeable during molt.

Birds may be subject to stronger selective pressure during energetically costly periods, such as molt, relative to other phases of their lifecycle. If some individuals of western passerine species are able to increase fitness or survival by tracking food resources to molt, it follows that selective pressure would favor these movements making them fairly common; perhaps much more common than is currently known. We believe that more long‐distance migrants have evolved these types of small‐scale movements in a greater proportion of species than resident and short‐distance migrant species. Further study examining molt migration strategies is needed to better understand the potential cascading effects of the molting period on population viability.

## CONFLICT OF INTEREST

None declared.

## AUTHOR CONTRIBUTIONS

Andrew Wiegardt and Dr. Jared Wolfe contributed to this manuscript during its conception, preliminary analysis, data collection, analysis, writing and revisions. Dr. C. J. Ralph contributed to this manuscript during conception, data collection, writing, revisions. Jaime Stephens and Dr. John Alexander contributed to this manuscript during the data collection, writing and revisions phases of this manuscript.

## Appendix A 1

### 1.1

A table summarizing the UTM coordinates (Zone 10), elevation, distance from the coast, state, number of captures, and region of each of the 80 banding stations located in northern California and southern Oregon

**Table:**

Station	NAD83 north	NAD83 east	Meters inland	Meters elevation	State	Total captures	Region
7MIL	4,728,473	575,844	190,427	1,279	OR	12,017	Upper Klamath Lake
ANT1	4,594,145	588,448	177,356	1,656	CA	8,821	Modoc County
APRI	4,682,417	480,646	87,084	352	OR	5,625	Rogue Basin
ASWA	4,660,998	526,897	127,315	1,631	OR	2,337	Siskiyou Mountains
BCBA	4,717,656	429,953	44,136	1,473	OR	3,548	Siskiyou Mountains
BIGS	4,500,266	629,450	221,772	1,401	CA	3,566	Modoc County
BRCR	4,701,578	506,756	118,679	479	OR	882	Rogue Basin
BUCK	4,661,697	538,572	139,004	837	OR	36	Rogue Basin
BURN	4,537,371	612,722	206,244	934	CA	2,292	Modoc County
CABL	4,744,004	372,231	112	54	OR	908	Coast
CABN	4,705,366	575,619	184,326	1,264	OR	20,808	Upper Klamath Lake
CAMP	4,571,740	453,222	43,829	119	CA	5,704	Klamath Trinity Rivers
CAPD	4,567,847	449,228	40,679	112	CA	7,310	Klamath Trinity Rivers
CLCR	4,483,853	550,587	151,481	140	CA	940	Inland Valley
DECC	4,680,570	447,272	67,654	391	OR	433	Rogue Basin
DOFL	4,630,428	442,441	39,518	1,207	CA	695	Siskiyou Mountains
EMMY	4,495,487	465,087	69,506	720	CA	1,967	Redwood Forest
GALB	4,301,943	478,483	24,811	291	CA	216	Redwood Forest
GBCR	4,666,497	465,477	67,654	656	OR	1,545	Rogue Basin
GERB	4,670,867	661,691	261,547	1,481	OR	4,876	Modoc County
GOLD	4,500,768	502,823	103,131	477	CA	60	Redwood Forest
GROV	4,534,016	459,081	53,161	1,258	CA	11,798	Trinity Mountains
HAMI	4,504,699	512,095	111,012	532	CA	3,119	Redwood Forest
HCME	4,692,750	445,024	61,362	864	OR	8,392	Rogue Basin
HOCK	4,510,523	494,318	92,449	437	CA	3,038	Redwood Forest
HOME	4,527,225	403,804	771	6	CA	47,304	Coast
HREC	4,316,491	493,628	45,802	305	CA	511	Redwood Forest
INVA	4,485,104	470,104	78,502	1,187	CA	10,962	Trinity Mountains
IVDO	4,485,275	469,570	77,948	1,177	CA	25	Trinity Mountains
JACR	4,521,540	409,649	8,276	3	CA	860	Coast
JENC	4,683,151	513,067	117,994	429	OR	1,972	Rogue Basin
JOHN	4,677,594	563,208	165,568	1,559	OR	8,303	Upper Klamath Lake
LADY	4,571,406	454,111	44,764	111	CA	5,523	Klamath Trinity Rivers
LELA	4,488,304	403,313	17,885	16	CA	2,441	Coast
LIAP	4,669,613	499,454	101,443	494	OR	484	Rogue Basin
LJON	4,676,958	562,711	164,968	1,549	OR	96	Upper Klamath Lake
LORI	4,675,632	409,356	22,295	498	OR	1,232	Rogue Basin
LOST	4,575,632	414,357	4,975	42	CA	488	Coast
MARI	4,522,302	416,714	14,582	33	CA	4,349	Coast
MAST	4,398,581	513,381	81,258	1,847	CA	5,174	High Elevation Mountains
NAVR	4,338,940	435,169	1,074	6	CA	1,046	Coast
NMTP	4,672,264	524,884	126,907	537	OR	1,103	Rogue Basin
NOMO	4,672,389	524,897	126,942	534	OR	499	Rogue Basin
NOMP	4,672,273	524,973	126,996	538	OR	14	Rogue Basin
ODES	4,698,000	577,166	183,697	1,263	OR	10,739	Upper Klamath Lake
ORCA	4,660,516	467,273	68,125	1,495	OR	2,858	Siskiyou Mountains
PARK	4,527,703	403,721	550	6	CA	7,533	Coast
PAST	4,527,646	403,986	821	3	CA	96	Coast
PCT1	4,632,371	482,449	78,664	409	CA	13,929	Rogue Basin
PLME	4,397,556	512,981	80,750	1,846	CA	4,215	High Elevation Mountains
QUIC	4,731,802	478,531	94,123	589	OR	2,095	Rogue Basin
REBA	4,499,162	503,843	104,573	490	CA	885	Redwood Forest
RECR	4,572,436	413,259	4,475	11	CA	2,628	Coast
RED2	4,568,039	449,395	40,811	110	CA	3,516	Klamath Trinity Rivers
SAC2	4,503,476	398,003	6,278	2	CA	405	Coast
SACR	4,503,476	398,007	6,282	2	CA	969	Coast
SBRR	4,502,936	507,146	106,673	509	CA	1,507	Redwood Forest
SCSP	4,653,482	510,549	110,471	2,087	OR	203	High Elevation Mountains
SFRD	4,500,347	503,279	103,690	475	CA	2,421	Redwood Forest
SIMS	4,545,895	553,700	147,201	502	CA	120	Modoc County
SKSW	4,701,442	549,463	158,259	1,096	OR	2,034	Upper Klamath Lake
SLEW	4,527,142	403,875	867	2	CA	299	Coast
SLOU	4,527,142	403,875	867	2	CA	1,647	Coast
SNBA	4,398,006	511,765	79,588	1,954	CA	116	High Elevation Mountains
SNCO	4,741,479	491,823	108,786	591	OR	3,119	Rogue Basin
SPEN	4,671,778	576,391	177,721	1,213	OR	276	Upper Klamath Lake
STFL	4,502,673	502,220	102,008	470	CA	95	Redwood Forest
SUCA	4,677,958	563,650	166,066	1,573	OR	115	Upper Klamath Lake
SVEN	4,507,778	516,408	114,453	550	CA	1,959	Redwood Forest
TAMO	4,462,166	572,146	154,296	93	CA	270	Inland Valley
TOPS	4,653,068	574,441	172,659	964	OR	11,746	Upper Klamath Lake
UHPO	4,500,867	505,752	105,905	535	CA	31	Redwood Forest
UNHI	4,500,867	505,752	105,905	535	CA	82	Redwood Forest
WEST	4,631,259	527,688	121,967	594	CA	554	Rogue Basin
WHBA	4,511,534	476,021	74,382	361	CA	2,248	Redwood Forest
WHSA	4,696,190	507,644	117,284	381	OR	1,455	Rogue Basin
WIIM	4,704,380	460,516	75,214	246	OR	25,763	Rogue Basin
WILL	4,723,290	593,905	207,471	1,276	OR	7,489	Upper Klamath Lake
WIWI	4,671,905	525,538	127,485	556	OR	12,061	Rogue Basin
WOOD	4,715,575	587,578	198,900	1,263	OR	9,101	Upper Klamath Lake
WREF	4,515,173	405,332	7,164	16	CA	3,167	Coast
YACR	4,490,470	410,385	23,273	49	CA	2,712	Coast

## Appendix B 1

### 1.1

Eighteen model selection tables examining the abundance of birds during the breeding and molting periods across a range of elevations and distances from the coast for nine species commonly captured in northern California and southern Oregon with AIC values, delta AIC values, number of parameters (*K*), *p*‐value of the beta estimate of the elevation or distance from the coast parameter, and AIC weights for each model. A “2” at the end of the model name denotes a quadratic model. All models included Julian date as a nuisance parameter.

**Table:**

Model	AIC	Delta AIC	*K*	*p*‐value	AIC weights
*Audubon's Yellow‐rumped Warbler Breeding*					
Region + Elevation2	10,562.3	0	13	<.0001	0.999
Region + Elevation	10,569.3	7	12	<.0001	0.0009
Region + Distance from Coast2	10,587.1	24.8	13	.079	<0.0001
Region	10,589.8	27.5	12	NA	<0.0001
Region + Distance from Coast	10,590.2	27.9	12	.2	<0.0001
Elevation2	10,632.6	70.3	3	<.0001	<0.0001
Distance from Coast	10,635.8	73.5	2	<.0001	<0.0001
Elevation	10,654.1	91.8	2	<.0001	<0.0001
Null	11,103.6	541.3	1	NA	<0.0001
*Audubon's Yellow‐rumped Warbler Molting*					
Region + Elevation2	3,999.1	0	13	.0012	0.875
Distance from Coast	4,001.9	2.8	2	<.0001	0.0532
Elevation2	4,002.1	3	3	<.0001	0.0435
Region + Distance from Coast	4,003.5	4.4	12	.094	0.0107
Region + Elevation	4,003.7	4.6	12	.011	0.0087
Region	4,003.9	4.8	12	NA	0.0072
Region + Distance from Coast2	4,005.5	6.4	13	.6	0.0014
Elevation	4,014.6	15.5	2	<.0001	<0.0001
Null	4,156.7	157.6	1	NA	<0.0001
*American Robin Breeding*					
Region + Elevation2	5,837.5	0	13	<.0001	0.9999
Region + Distance from Coast	5,868	30.5	12	<.0001	<0.0001
Region + Distance from Coast2	5,870	32.5	13	.18	<0.0001
Region	5,889.1	51.6	12	NA	<0.0001
Region + Elevation	5,891.1	53.6	12	.91	<0.0001
Elevation2	6,098	260.5	3	<.0001	<0.0001
Elevation	6,149	311.5	2	<.0001	<0.0001
Distance from Coast	6,397.3	559.8	2	<.0001	<0.0001
Null	6,440.5	603	1	NA	<0.0001
*American Robin Molting*					
Region + Elevation2	3,999.1	0	13	.0012	0.8750
Distance from Coast	4,001.9	2.8	2	<.0001	0.0532
Elevation2	4,002.1	3	3	<.0001	0.0435
Region + Distance from Coast	4,003.5	4.4	12	.094	0.0107
Region + Elevation	4,003.7	4.6	12	.110	0.0087
Region	4,003.9	4.8	12	NA	0.0072
Region + Distance from Coast2	4,005.5	6.4	13	.61	0.0014
Elevation	4,014.6	15.5	2	<.0001	<0.0001
Null	4,156.7	157.6	1	NA	<0.0001
*Orange‐crowned Warbler Breeding*					
Region + Elevation	6,081.5	0	12	.0001	0.6899
Region + Elevation2	6,082.3	0.8	13	.45	0.3100
Elevation	6,092.1	10.6	2	<.0001	<0.0001
Elevation2	6,093.4	11.9	3	.0039	<0.0001
Region	6,098.8	17.3	12	NA	<0.0001
Region + Distance from Coast2	6,099.9	18.4	13	.079	<0.0001
Region + Distance from Coast	6,100.7	19.2	12	.830	<0.0001
Distance from Coast	6,118.7	37.2	2	<.0001	<0.0001
Null	6,220.3	138.8	1	NA	<0.0001
*Orange‐crowned Warbler Molting*					
Region + Distance from Coast2	4,957.5	0	13	.035	0.9994
Region + Distance from Coast	4,965.1	7.6	12	.001	0.0005
Region + Elevation2	4,974	16.5	13	.025	<0.0001
Region	4,974.8	17.3	12	NA	<0.0001
Region + Elevation	4,976.4	18.9	12	.52	<0.0001
Elevation2	5,013.5	56	3	<.0001	<0.0001
Elevation	5,019.3	61.8	2	<.0001	<0.0001
Distance from Coast	5,051.9	94.4	2	<.0001	<0.0001
Null	5,160	202.5	1	NA	<0.0001
*Oregon Dark‐eyed Junco Breeding*					
Region + Elevation2	10,443.9	0	13	<.0001	0.9999
Region + Elevation	10,506.8	62.9	12	<.0001	<0.0001
Region + Distance from Coast2	10,594	150.1	13	.0265	<0.0001
Region	10,595.2	151.3	12	NA	<0.0001
Region + Distance from Coast	10,597.1	153.2	12	<.0001	<0.0001
Elevation	10,799	355.1	2	<.0001	<0.0001
Elevation2	10,800.9	357	3	<.0001	<0.0001
Distance from Coast	11,259.7	815.8	2	<.0001	<0.0001
Null	11,496.9	1,053	1	NA	<0.0001
*Oregon Dark‐eyed Junco Molting*					
Region + Elevation2	8,706.4	0	13	<.0001	0.9999
Region + Elevation	8,928.3	221.9	12	<.0001	<0.0001
Elevation2	9,009.9	303.5	3	<.0001	<0.0001
Region + Distance from Coast2	9,018.3	311.9	13	.0049	<0.0001
Region	9,024.7	318.3	12	NA	<0.0001
Region + Distance from Coast	9,025.8	319.4	12	.35	<0.0001
Elevation	9,065.6	359.2	2	<.0001	<0.0001
Distance from Coast	9,439.1	732.7	2	<.0001	<0.0001
Null	9,666.4	960	1	NA	<0.0001
*MacGillivray's Warbler Breeding*					
Region + Elevation2	10,858	0	13	<.0001	0.9999
Region + Elevation	10,896.2	38.2	12	<.0001	<0.0001
Region + Distance from Coast2	10,972.4	114.4	13	.051	<0.0001
Region + Distance from Coast	10,990.5	132.5	12	<.0001	<0.0001
Elevation2	11,057	199	3	<.0001	<0.0001
Region	11,074.6	216.6	12	NA	<0.0001
Elevation	11,129.1	271.1	2	<.0001	<0.0001
Distance from Coast	11,267.3	409.3	2	<.0001	<0.0001
Null	11,478.9	620.9	1	NA	<0.0001
*MacGillivray's Warbler Molting*					
Region + Elevation2	4,799.7	0	13	<.0001	0.9992
Region + Elevation	4,807.36	7.66	12	.0001	0.0004
Region + Distance from Coast	4,808.2	8.5	12	.0001	0.0002
Region + Distance from Coast2	4,809.9	10.2	13	.194	<0.0001
Elevation2	4,820.6	20.9	3	<.0001	<0.0001
Region	4,820.8	21.1	12	<.0001	<0.0001
Elevation	4,850.3	50.6	2	.0310	<0.0001
Distance from Coast	4,855	55.3	2	.85	<0.0001
Null	4,912.7	113	1	<.0001	<0.0001
*Song Sparrow Breeding*					
Region + Distance from Coast2	24,500	0	13	.0001	0.9999
Region + Distance from Coast	24,537.6	37.6	12	<.0001	<0.0001
Region + Elevation2	24,570	70	13	.0002	<0.0001
Region + Elevation	24,578.2	78.2	12	0.039	<0.0001
Region	24,580.4	80.4	12	NA	<0.0001
Distance from Coast	24,740.4	240.4	2	<.0001	<0.0001
Elevation	24,819	319	2	<.0001	<0.0001
Elevation2	24,819.4	319.4	3	<.0001	<0.0001
Null	25,616.8	1,116.8	1	NA	<0.0001
*Song Sparrow Molting*					
Region + Distance from Coast2	16,900.3	0	13	.0177	0.9999
Region + Distance from Coast	16,915.5	15.2	12	<.0001	<0.0001
Region	16,930	29.7	12	NA	<0.0001
Region + Elevation	16,931.9	31.6	12	.707	<0.0001
Region + Elevation2	16,932.3	32	13	.184	<0.0001
Distance from Coast	16,975.1	74.8	2	<.0001	<0.0001
Elevation2	17,006.6	106.3	3	<.0001	<0.0001
Elevation	17,012.4	112.1	2	<.0001	<0.0001
Null	17,361.5	461.2	1	NA	<0.0001
*Spotted Towhee Breeding*					
Region + Elevation2	9,399.6	0	13	<.0001	0.9999
Region + Distance from Coast2	9,453	53.4	13	.001	<0.0001
Region + Distance from Coast	9,465.5	65.9	12	.0042	<0.0001
Region	9,471.9	72.3	12	NA	<0.0001
Region + Elevation	9,472.7	73.1	12	.0430	<0.0001
Elevation2	9,735.1	335.5	3	<.0001	<0.0001
Elevation	9,779.7	380.1	2	<.0001	<0.0001
Distance from Coast	9,795.8	396.2	2	.013	<0.0001
Null	9,931.7	532.1	1	NA	<0.0001
*Spotted Towhee Molting*					
Region + Elevation2	4,735.1	0	13	.0004	0.9999
Region + Distance from Coast2	4,748.6	13.5	13	.0073	<0.0001
Region + Elevation	4,753.1	18	12	.088	<0.0001
Region	4,754.1	19	12	NA	<0.0001
Region + Distance from Coast	4,754.8	19.7	12	.26	<0.0001
Elevation2	4,831.5	96.4	3	<.0001	<0.0001
Distance from Coast	4,877.1	142	2	.0008	<0.0001
Elevation	4,878.7	143.6	2	.0021	<0.0001
Null	5,148	412.9	1	NA	<0.0001
*Swainson's Thrush Breeding*					
Region + Distance from Coast2	11,071.4	0	13	<.0001	0.9999
Region + Elevation2	11,176.7	105.3	13	<.0001	<0.0001
Region + Elevation	11,225.8	154.4	12	<.0001	<0.0001
Region	11,243	171.6	12	NA	<0.0001
Region + Distance from Coast	11,243.6	172.2	12	.225	<0.0001
Distance from Coast	11,601	529.6	2	<.0001	<0.0001
Elevation2	11,626.1	554.7	3	<.0001	<0.0001
Elevation	11,652.4	581	2	<.0001	<0.0001
Null	12,282.8	1,211.4	1	NA	<0.0001
*Swainson's Thrush Molting*					
Region + Distance from Coast2	4,234.8	0	13	<.0001	0.9998
Region + Elevation2	4,243.4	8.6	13	.0737	0.0001
Region + Elevation	4,255.4	20.6	12	<.0001	<0.0001
Region + Distance from Coast	4,266	31.2	12	.0015	<0.0001
Region	4,273.2	38.4	12	NA	<0.0001
Distance from Coast	4,281.7	46.9	2	<.0001	<0.0001
Elevation	4,292.6	57.8	2	.0006	<0.0001
Elevation2	4,294.6	59.8	3	.39	<0.0001
Null	4,362.1	127.3	1	NA	<0.0001
*Wrentit Breeding*					
Region + Distance from Coast2	8,239.1	0	13	0.0076	0.9999
Region + Distance from Coast	8,267	27.9	12	<.0001	<0.0001
Region + Elevation2	8,271.4	32.3	13	.0051	<0.0001
Region + Elevation	8,272.9	33.8	12	.0005	<0.0001
Region	8,283.9	44.8	12	NA	<0.0001
Elevation2	8,348.4	109.3	3	.0001	<0.0001
Elevation	8,387.2	148.1	2	.003	<0.0001
Distance from Coast	8,394.6	155.5	2	.120	<0.0001
Null	8,413.1	174	1	NA	<0.0001
*Wrentit Molting*					
Region + Elevation	3,184.9	0	12	<.0001	0.8581
Region + Elevation2	3,186.7	1.8	13	.001	0.1418
Elevation2	3,204.9	20	3	.037	<0.0001
Elevation	3,205.8	20.9	2	<.0001	<0.0001
Region + Distance from Coast2	3,218.7	33.8	13	.0008	<0.0001
Region	3,234.7	49.8	12	NA	<0.0001
Region + Distance from Coast	3,235	50.1	12	.21	<0.0001
Distance from Coast	3,252.4	67.5	2	.0013	<0.0001
Null	3,437.8	252.9	1	NA	<0.0001
